# Review of cancer therapies for the perioperative physician

**DOI:** 10.1186/s13741-023-00315-1

**Published:** 2023-06-13

**Authors:** Anahita Dabo-Trubelja, Vijaya Gottumukkala

**Affiliations:** 1 Department of Anesthesiology and Critical Care, Onco-Anesthesia Fellowship, Perioperative Echocardiography and Ultrasound, Memorial Sloan Kettering Cancer Center of Weill Cornell Medical Center, 1274 York Ave C-330, New York, NY 10065 USA; 2 Department of Anesthesiology and Perioperative Medicine Program for Advancement of Perioperative Cancer Care, MD Anderson Cancer Center, The University of Texas, Houston, TX USA

**Keywords:** Perioperative physician, Anesthesiology, Chemotherapy, Radiation therapy, Immunotherapy, Cancer vaccines, Interventional radiology therapy

## Abstract

Advances in cancer treatments over the past decades combining chemotherapy with novel technologies in immunotherapies, radiation therapies, and interventional radiology have prolonged life expectancy. Patients have more options for treatments of their primary or metastatic diseases. Increased procedural techniques amid an aging population with multiple comorbidities present risks and challenges in the perioperative period.

Chemotherapy remains the mainstay of cancer treatment, can be given intraoperatively, and is combined with other treatment modalities. Immunotherapy is particular to cancer cells while being less toxic to healthy cells. Cancer vaccines stimulate the immune system to stop disease progression. Oncolytic viruses enhance the immune system’s cytotoxic effect and show promise to halt metastatic disease progression if present in the perioperative period. Novel techniques in radiation therapy combined with traditional treatments show enhanced survival. This review focuses on current cancer treatments encountered in the perioperative period.

## Background

The past several decades introduced novel therapeutic modalities and surgical techniques in the fight against cancer. The growing periprocedural and perioperative needs of patients with active disease and survivors alike compel an understanding of new systemic therapies and their side effects. This review will focus on current chemotherapy, immunotherapies, radiation therapies, interventional radiology, and their side effect profile and concerns in the perioperative period.

## Chemotherapeutic agents

In 1891, the German Chemist Paul Ehrlich was the first to use chemicals to treat diseases. Over the following decades, the ensuing discovery of different chemotherapeutic agents and combination chemotherapy remained the mainstay of cancer treatment. Chemotherapy is administered preoperatively as neoadjuvant chemotherapy to alleviate tumor burden during surgery: during the perioperative period as adjuvant chemotherapy to decrease the risk of cancer recurrence and palliative chemotherapy to improve quality of life and prolong survival when there is no chance of cure (DeVita and Chu 2008). Systemic chemotherapy is also associated with long‐term toxicities (Adam et al. 2009). The future of conventional chemotherapy alone or as part of a combined regimen lies in patient‐specific benefit vs. risk of treatment‐related long‐term toxicity, recurrence of disease, or death.

Chemotherapeutic agents interfere with the metabolic processes of DNA, RNA, and protein synthesis of the cancer cell. They target cancer cells in two ways: at different cell cycle phases and boost the host immune response by activating various lymphocyte cell receptors (TLR‐4, CD4, CD8) to re‐establish immunosurveillance (Galluzzi et al. 2015).

Classic chemotherapeutic agents are categorized according to their chemical nature and function:

1. Alkylating agents replace the alkyl group with hydrogen atoms in DNA so the cancer cells cannot repair.
2. Plant alkaloids bind to microtubule proteins during the mitotic phase and inhibit replication.
3. Antimetabolites deplete nucleotides inhibiting DNA replication of the cancer cells.
4. Antitumor antibiotics inhibit enzymes required for DNA synthesis.
5. Topoisomerase inhibitors inhibit the topoisomerase enzyme, so the cancer cell cannot replicate.
6. Corticosteroids are anti‐inflammatory and inhibit protein synthesis.

These drugs are not tumor specific and damage healthy cells as well. They are used alone or combined with other agents or treatments (Gehdoo 2009). Table 1 shows the cell cycle’s classification, mechanism of action, targeted cancer, and toxicities.

**Table 1:** Classification, mechanism of action, most common side effects of chemotherapeutic agents, and targeted cancers

Class	Drug	Mechanism of action	Targeted cancer	Common side effects
Alkylating agent	**Nitrogen mustards** — chlorambucil, cyclophosphamide, ifosfamide, chlormethine, melphalan **Alkylsulfonates** — busulfan **Nitrosoureas** — streptozocin, carmustine, lomustin **Triazines** — dacarbazine Ethylenimines — thiotepa, altretamine **Platinums** — cisplatin, carboplatin, oxaliplatin **Hydrazine** — methylhydrazine-benzamine, procarbazine	Replace the alkyl group with hydrogen atoms in DNA so the cancer cells cannot repair	Leukemia, multiple myeloma, sarcoma, lung, breast, and ovarian cancer. Nitrosoureas cross the blood‐brain barrier useful for treating brain tumors	Bone marrow suppression, dose‐dependent risk of leukemia, renal failure, liver failure, cardiomyopathy, pulmonary fibrosis, peripheral neuropathy
Antimetabolites	**Folic acid antagonist** — methotrexate, pemetrexed, praxatrexate **Pyrimidine antagonist** — 5‐fluorouracil (5‐FU), decitabine, floxuridine, capecitabine, gemcitabine, cytarabine **Purine antagonist** — 6-mercaptopurine, 6-thioguanine **Adenosine deaminase inhibitor** — cladribine, fludarabine, nelarabine, pentostatin, hydroxyurea, nelarabine, clofarabine, azacytidine	Deplete nucleotides inhibiting DNA replication of the cancer cells	Leukemia, breast, ovarian, gastrointestinal tract, lung, pancreatic, bladder, sarcoma, head, and neck cancers	Pancytopenia, increase in liver function test, kidney failure,‐pulmonary edema, cardiac toxicity
Antitumor antibiotics	**Anthracyclines** — doxorubicin, daunorubicin, idarubicin, epirubicin, valrubicin **Non‐anthracyclines** — bleomycin, dactinomycin, mitomycin	Antibiotics that inhibit enzymes required for DNA synthesis	Breast, ovarian, prostate, germ cell, sarcoma, lung, liver, bladder, thyroid, lymphoma, leukemia, neuroblastoma, Wilms	Cardiomyopathy, pulmonary fibrosis, renal, and hepatobiliary toxicity
Topoisomerase inhibitors	**Type 1** (camptothecins) — topotecan, irinotecan **Type 2** (epipodophyllotoxin s) —etoposide, teniposide, mitoxantrone	Inhibit the enzyme topoisomerase — which inhibits DNA synthesis	Leukemia, lung, ovarian, GI tract, colon, pancreatic	Type 2 increases the risk of second cancer
Plant alkaloids	**Taxanes** — placitaxel, docetaxel **Vincas** — vinblastine, vincristine, vinorelbine	Inhibit mitosis	Breast, lung, multiple myeloma, leukemia, lymphoma	Dose-dependent nerve damage Pulmonary Cardiac and hepatobiliary toxicity
Corticosteroids	Prednisone, dexamethasone methylprednisolone	Anti‐inflammatory inhibits protein synthesis	Leukemia, lymphoma, multiple myeloma	Adrenal insufficiency, myopathy, decreased wound healing

### Perioperative period

Evaluation of different organ systems allows for the preoperative assessment of chemotherapy-induced organ dysfunction (Table 2). A detailed history of cancer management is essential. Changes in a therapeutic dose and time interval, side effects, or intolerance towards a specific organ system necessitating a different chemotherapeutic agent indicate concerns for the organ affected. Diminished organ and physiologic reserve increase the risk for interactions between anesthetic agents and specific anticancer therapies, causing uncertain or unpredictable life-threatening complications.

**Table 2:** Organ system toxicity and the associated chemotherapeutic agent

Organ system toxicity	Chemotherapeutic agent
Pulmonary	Antitumor antibiotics, alkylating agents, antimetabolites, plant alkaloids
Cardiovascular	Antitumor antibiotics (anthracyclines and mitomycin), plant alkaloids, alkylating agents (cyclophosphamide, ifosfamide, cisplatin, busulfan, carmustine, chlormethine), antimetabolite (5‐FU)
Hepatobiliary	Alkylating (nitrosoureas, platinums, nitrogen mustards) antimetabolites (5-FU), antitumor antibiotics, plant alkaloids
Renal	Antitumor antibiotics, antimetabolites, alkylating agents

#### Time interval

The time interval between the last cycle of chemotherapy and surgery is not well defined. The perioperative tumor microenvironment stimulates oncogenic growth factors and reduces the immunosuppressive activities of interleukin-2 and lymphokine-activated killer cells, facilitating the spread of metastasis. Ideally, postsurgical time to chemotherapy should be brief. Current recommendations are from retrospective analyses of breast and colorectal cancer patients. Adjuvant chemotherapy started within 20 days of surgery significantly improved disease-free survival in breast cancer patients (Sanford et al. 2016). A recent meta-analysis found an increased mortality risk in colorectal and gastric cancer with adjuvant chemotherapy delayed greater than 8-week post-surgery. The maximum benefit in survival is within 6–8 weeks of colorectal surgery. In contrast, lung cancer and pancreatic cancer showed no difference. The high mortality risk in postsurgical time to chemotherapy was an independent variable compared to other clinical and histopathological characteristics (Petrelli et al. 2019).

#### Immunologic effect

Chemotherapeutic agents affect both the innate and adaptive immune systems. The innate system response is myelosuppression reflected in a pancytopenia which can last a few weeks. If severe, systemic infection and sepsis risk increase. Preoperative tests include hemoglobin, thrombocyte, platelet, and white blood cell count with differential. Optimization before surgery with multiple transfusions, antibiotics, and granulocyte-stimulating factors to boost neutrophil production is sometimes necessary (Huettemann and Sakka 2005). A recent analysis showed that in patients undergoing emergency laparotomy, a leukocyte count < 4.0 × 10 (9)/L was associated with increased perioperative mortality but was not prohibitive even after adjusting for other patient-related factors (Gulack et al. 2015).

Chemotherapy induces an inflammatory response responsible for decreasing anticoagulant factors leading to a procoagulant state. A current systematic review of patients undergoing cytoreductive surgery with hyperthermic intraperitoneal chemotherapy for colorectal cancer metastasis noted a low risk for postoperative bleeding and thrombotic events after 30 and 90 days, respectively (Lundbech et al. 2022).

#### Cardiovascular

Cardiac toxicities manifest at various times during and after treatment. An underlying cardiac abnormality becomes unmasked under general anesthesia, even in patients with noted normal left ventricular function (Huettemann et al. 2004). A comprehensive preoperative evaluation examines the patient for signs and symptoms of cardiac toxicity and obtains routine EKG, troponin, and B-natriuretic peptide (BNP) levels. A cardiology evaluation and optimization before surgery are needed if cardiotoxicity is suspected. Anthracyclines are known to cause cardiomyopathy and QTc prolongation. Prophylactic use of carvedilol and angiotensin-converting enzymes prevents left ventricular ejection fraction decline and diastolic dysfunction in patients receiving anthracycline chemotherapy (Abuosa et al. 2018; Avila et al. 2018).

Cardiomyopathy is also associated with bleomycin, cyclophosphamide, and busulfan therapy. Paclitaxel, and cisplatin, in combination, infrequently produces unanticipated side effects such as ventricular tachycardia. 5‐Fluorouracil (5FU) is associated with coronary vasospasm and could complicate the differential diagnosis of chest pain in the postoperative period (Svanström et al. 2018). Intraoperatively, avoid drugs that cause QTc prolongation. Invasive monitoring is often required depending on the extent of surgery and patient factors. Postoperatively, the patient may require admission to the intensive care unit for further management and care.

#### Pulmonary

Pulmonary toxicity from bleomycin therapy is well known. It usually occurs 10 weeks after treatment. The production of free radicals and superoxide is a side effect of bleomycin toxicity: 40% will develop pulmonary toxicity, 30% pulmonary fibrosis within this group, and 10% ARDS-related mortality. The risk increases if the patient receives cisplatin therapy. Free radical and superoxide production increase with a higher concentration of inspired oxygen. Therefore, inspired oxygen concentration during general anesthesia is kept at or below 30% to maintain oxygen saturation between 89 and 92%. However, controversial and conflicting data exist. Some authors suggest restrictive fluid management is more important than the inspired oxygen concentration (Aakre et al. 2014). Years after bleomycin therapy, a high concentration of inspired oxygen concentration during the perioperative period may provoke pulmonary toxicity even in asymptomatic patients.

The alkylating agent mitomycin C can lead to respiratory distress. Antimetabolites, gemcitabine, and methotrexate can cause diffuse alveolar hemorrhage, noncardiac pulmonary edema, and pleural effusions. Weeks after platinum compounds paclitaxel and docetaxel therapy, interstitial pneumonitis may develop. The patient may present with a chronic cough and dyspnea before surgery with bibasilar crackles, wheezing, or coarse breath sounds on lung auscultation. Preoperative evaluation should include baseline oxygen saturation and chest X-ray. Pulmonary function testing, including carbon monoxide diffusion capacity (DLCO), is recommended to detect occult changes in pulmonary function and optimize the patient before surgery (Kessell et al. 2009). A CT scan is sensitive to parenchymal lung changes; however, its prognostic value remains uncertain. Pulmonary toxicity is a diagnosis of exclusion, and patients receive glucocorticosteroid therapy. Intraoperative management includes lung-protective ventilation and stress dose steroids (Leger et al. 2017).

#### Renal

The kidneys excrete chemotherapeutic agents, which can lead to acute kidney injury and renal failure. Most are dose-related injuries and reversible. Cisplatin treats many malignancies and may lead to acute kidney injury (AKI) or chronic kidney disease (CKD), especially during cytoreduction surgery with intraoperative hyperthermic intraperitoneal chemotherapy (HIPEC). Multiple mechanisms of action lead to renal toxicity; decreased renal blood flow from vasoconstriction of the kidney vasculature; and increased proinflammatory cytokines, proximal tubule, and loop of Henle cellular necrosis. Acute kidney injury manifests as early as 5 to 7 days after therapy or even earlier in patients with preexisting kidney disease. The elderly, hypoalbuminemia, female, smoking, a history of hypertension, a total cisplatin dose > 100 mg, concomitant paclitaxel, or other nephrotoxic drugs are also risk factors. Clinical evidence of a decrease in filtration rate or a rise in creatinine level to 1.5–2.9 limits the dose administered. Ifosfamide can cause Fanconi syndrome 48 months after therapy (Nicolaysen 2020). Preoperative evaluation includes serum electrolytes, urea, creatinine, and glomerular filtration rate. Patients with preexisting kidney disease should have adjusted doses of drugs excreted by the kidneys, including antibiotics and opioids. Prevention is best achieved with isotonic saline to maintain diuresis above 100 ml/h. In HIPEC, some protocols involve administering sodium thiosulfate (Naffouje et al. 2018).

#### Hepatic

The liver metabolizes most chemotherapeutic agents, and patients may present for surgery with asymptomatic liver disease. The antimetabolite, 5-FU, and alkylating agents cause steatosis, increasing the risk of perioperative blood loss. Platinum compounds lead to hepatic necrosis. Preoperative liver function test measurements of alkaline phosphatase, bilirubin, glutamyl transpeptidase, alanine aminotransferase, and international normalized ratio (INR) evaluate liver function. General anesthesia is usually tolerated well. If hepatic dysfunction is apparent in laboratory measures, it contributes to prolonged anesthesia recovery. In addition, the effect of decreased hepatic blood flow under general anesthesia contributes to prolonged recovery. Neuraxial and regional anesthesia have a lower systemic effect, do not delay awakening from anesthesia, and should be used whenever possible (Rahimzadeh et al. 2014).

#### Neurological

Central or peripheral neurotoxicity is a common side effect of chemotherapy. It is dose related and commonly seen in patients with diabetes mellitus, advanced age, and preexisting neuropathies. Central neurotoxicity has a broad spectrum of symptoms, from encephalopathy to hemiparesis and progressive dementia. Peripheral neurotoxicity manifests as peripheral neuropathy. Symptoms start with the onset of chemotherapy and stabilize during treatment. The vinca alkaloids and the platinum-alkylating agents can worsen an underlying neuropathy. Preoperatively, any preexisting abnormalities are documented. Regional anesthesia is not contraindicated. Autonomic dysfunction may present as orthostatic hypotension or vasovagal episodes, especially in the postoperative period (Was et al. 2022).

#### Gastrointestinal

Diarrhea is a side effect of many cancer drugs leading to serum electrolyte disturbances and fluid abnormalities. Optimal intravascular status is essential for improved outcomes after surgery. Preoperatively, signs of intravascular depletion may require intraoperative monitoring of fluid status. Various noninvasive or invasive hemodynamic monitors of pulse pressure variation (PPV), stroke volume variation (SVV), and systolic blood pressure variation (SBPV) are available. Inferior vena cava diameter variation using ultrasound guidance is also beneficial, and concurrent vasopressor infusion for blood pressure support may be needed until the optimal fluid state is achieved (Marik et al. 2009).

#### Medications

Some commonly used medications in the perioperative period interact with chemotherapeutic agents. Significant anticholinesterase activity by cyclophosphamide lasts 3–4 weeks after administration. A reduction in succinylcholine dose prevents prolonged neuromuscular blockade and respiratory depression. Procarbazine, a monoamine oxidase inhibitor, and ephedrine, an indirect sympathomimetic, facilitate an exaggerated blood pressure response (Armand et al. 2007). Nonsteroidal anti‐inflammatory drugs (NSAIDs) compete for the same receptor site at the renal tubule and reduce the excretion of methotrexate, which may lead to fatal side effects. During HIPEC, ondansetron reduced the plasma concentration of cisplatin, and furosemide can add to its toxicity (Naffouje et al. 2018).

## Immunotherapy

Cancer immunotherapy activates the host’s antitumor immune response in different ways. It includes molecular and cell-targeted therapy. Molecular-targeted therapy acts on molecules such as surface antigens, growth factors, receptors, and signal transduction pathways that regulate the cell cycle, progression of the disease, or death. Thus, it is sometimes called a monoclonal antibody, check-point inhibitor, precision therapy, or personalized medicine (Røsland and Engelsen 2015). Cell-based therapies are genetically manipulated T-cell lymphocytes with T-cell receptors that recognize tumor-specific antigens and are called cell‐based immunotherapy, adaptive cell therapy (ACT), or chimeric antigen receptor T-cell (CAR T cells) therapy.

### Molecular-targeted therapy — small or large monoclonal antibody or check-point inhibitor

Molecular-targeted therapy has two families: a small and a large monoclonal antibody molecule called a check-point inhibitor. A small monoclonal antibody penetrates the cells to target specific proteins inside the cell. They are approved to treat over 15 cancer types and are recognizable because they all end in ‐ib. Table 3 shows the types of small molecule targeted therapy, stem ending and name, the intended target, and side effects (Lee et al. 2018).

**Table 3:** Classification of small molecule target therapy (‐ibs), intracellular target, and side effects

Small molecule	End with name	Target	Target action	Side effect
Tyrosine kinase inhibitors	‐tinib over 50 FDA approved	EGFR, VEGFR, PDGFR, FGFR, SCGFR, ALK, ERBB2, ITK, LCK, BTK, CDK	Inhibit cell function, growth factors, proliferation, angiogenesis	High blood pressure, bleeding, thrombosis, periorbital edema, cardiomyopathy
Proteasome inhibitors	‐zomib Bortezomib (Velada) Carfilzomib (Kyprolis) Ixazomib (Ninlaro)	Proteasome	Apoptosis‐cell death	Pancytopenia, peripheral neuropathy, diarrhea
Cyclin‐dependent inhibitors (CDK)	‐ciclib Abemaciclib (Verzenio) Palbociclib (Ibrance) Ribociclib (Kisqali)	CDK4/6‐HER2	DNA synthesis‐cell death	Bone marrow suppression
Poly‐ADP‐ribosome polymerase inhibitors (PARP)	‐parib Olaparib (Lynparza) Niraparib (Zejula) Rucaparib (Rubraca) Talazoparib (Talzenna)	PARP‐BRACA1, BRACA2	Inhibits DNA repair‐cell death	Bone marrow suppression, MDS, AML, HTN, hypertensive crisis

*EGFR* Epidermal growth factor receptor, *VEGFR* Vasoendothelial growth factor receptor, *PDGFR* Platelet‐derived growth factor receptor, *FGFR* Fibroblast growth factor, *SCGFR* Stem cell growth factor, *ALK* Anaplastic lymphoma kinase, *ERBB2* Breast cancer, *ITK* Interleukin‐2 receptor, *LCK* Leukocyte-specific protein kinase, *BTK* Bruton‐B-cell‐specific kinase, *CDK* Cyclic-dependent kinase, *HER2* Breast cancer receptor, *BRACA1, BRACA2* Breast cancer gene, *MDS* Myelodysplastic syndrome, *AML* Acute myeloid leukemia, *HTN* Hypertension

Large molecular monoclonal antibodies or check-point inhibitors bind to surface proteins to inhibit tumor growth by direct or indirect action. Immediate action includes the following: (1) binding to an antigen, cell receptor, or membrane protein disrupts the signaling pathway, inhibiting cell proliferation and activating cell death, (2) acting as a carrier to deliver toxins inducing cell death, and (3) inducing apoptosis by (1) complement‐dependent cytotoxicity (CDC), (2) classic complement cascade leading to cell lysis, (3) antibody‐dependent cell‐mediated cytotoxicity (ADCC), and (4) recruiting natural killer cells (NK), macrophages, or monocytes (Weiner et al. 2010). Large monoclonal antibody drugs or check-point inhibitors are recognizable because they all end in -mab. The most used to treat cancer are shown in Table 4 (Baldo 2016). The side effect profile of monoclonal antibodies is extensive and often limits therapeutic effect. The most pronounced is cytokine release syndrome (fever, nausea, headache, tachycardia, hypotension, and acute dyspnea), often severe enough to warrant intensive treatment.

**Table 4:** Classification of extensively targeted immunotherapy (‐mab) by approved indication, target malignant cell receptor, and mechanism of action

Cancer	Name	Trade name	Target cell receptor	Mechanism of action
Multiple myeloma	Elotuzumab Daratumumab	Empliciti Darzalex	SLAMF7 CD38	NK, ADCC Apoptosis, ADCC
Acute lymphocytic leukemia	Blinatumomab	Blincyto	CD19/CD3	Granzyme and perforin cell-induced death
Chronic lymphocytic leukemia	Rituximab Alemtuzumab Obinutuzumab Ofatumumab	Rituxan Campath Gazeva Arzerra	CD20 CD52 CD20 CD20	ADCC ADCC ADCC ADCC
Non‐Hodgkin’s lymphoma	Ibritumomab	Zevalin	CD20	B‐emission cell death
Stem cell transplant failure	Brentuximab	Adcetris	CD30	Apoptosis
Colorectal	Cetuximab Bevacizumab Panitumumab Ramucirumab	Erbitux Avastin Vectibix Cyramza	EGFR VEGF‐A EGFR VEGFR2	Blocks phosphorylation Inhibits angiogenesis & metastatic disease
Head & neck	Cetuximab	Erbitux	EGFR	Blocks phosphorylation
Lung	Bevacizumab Pembrolizumab Necitumumab Nivolumab Ramucirumab Atezolizumab	Avastin Keytruda Portrazza Opdivo Cyramza Tecentriq	VEGF‐A PD‐1 EGFR PD‐1 VEGFR2 PD‐1, CD80	Inhibits angiogenesis Apoptosis Prevents inhibition of antitumor immune response Antitumor growth
Breast	Bevacizumab Pertuzumab Trastuzumab Ado‐trastuzumab Pembrolizumab Atezolizumab Avelumab Durvalumab	Avastin Perjeta Herceptin Kadcyla Keytruda Tecentriq Bavencio Infinzi	VEGF‐A HER2 HER2 HER2 PDL-1 PDL-1 PDL-1 PDL-1	Inhibits angiogenesis Apoptosis ADCC Apoptosis
Ovarian, peritoneal, glioblastoma	Bevacizumab	Avastin	VEGF‐A	Inhibits angiogenesis & metastatic disease
Melanoma	Pembrolizumab Ipilimumab Nivolumab	Keytruda Yervoy Opdivo	PD‐1 CTLA‐4 PD‐1	Prevents inhibition of antitumor immune response
Gastric	Trastuzumab Ramucirumab	Herceptin Cyramza	HER2 VEGFR2	ADCC Inhibits angiogenesis
Solid tumor bone metastasis	Denosumab	Prolia Xgeva	RANKL	Antitumor growth
Urothelial	Atezolizumab	Tecentriq	PD‐1, CD80	Antitumor growth
Malignant ascites	Catumaxomab	Removab	CD3	T-cell-induced death

*NK* Natural killer cells, *ADCC* Antibody-dependent cell‐mediated cytotoxicity

Molecular-targeted immunotherapy is often combined with other modalities and may last for years. A study on lung cancer patients looked at the enhanced effects of molecular immunotherapy in the perioperative setting. Results favor combining these two treatment modalities to improve cancer outcomes (Forde et al. 2018). The greatest challenge for the future of molecular-targeted therapy is the heterogenicity and the associated resistance that develops within the tumor. The development of new targeted therapies is picking up as new targets are found (Suzuki et al. 2015). Together with other treatment modalities, molecular-targeted therapy lays the foundation for a precision approach to cancer treatment based on a patient’s genetic profile.

#### Perioperative period

In the perioperative period, the patient may exhibit signs of endocrine, cardiac, and pulmonary toxicities. The most common is an inflamed pituitary gland, clinically presenting as hypothyroidism, primary adrenal insufficiency, or diabetes insipidus. The patient should have any endocrine test related to organ dysfunction which occurred after the onset of treatment (e.g., adrenocorticotropic hormone (ACTH), hemoglobin A1c (HbA1c), thyroid function tests) and a consultation with an endocrinologist (Lewis et al. 2020). Cardiac manifestations of myocarditis present as fatigue, dyspnea, and chest pain. Preoperative workup includes brain natriuretic peptide, troponin, EKG, and a consultation with a cardiologist. Pulmonary toxicity is rare, but chronic cough, dyspnea, and chest pain symptoms merit a pulmonary consultation to differentiate it from other chronic pulmonary diseases. Antibiotics and stress doses of steroids may be needed in the perioperative period. Commonly encountered molecular-targeted therapies deserve mention. Bevacizumab (Avastin), used in metastatic colorectal cancer, is known to decrease wound healing and causes thromboembolic events. It should be discontinued 6–8 weeks before surgery and restarted > 28 days after surgery (Libert et al. 2010). Herceptin (trastuzumab), indicated in HER2 + metastatic breast cancer, is associated with cardiac toxicity. Cardiac toxicity increases with previous radiation therapy, negative receptor status, high BMI, low baseline LVEF, and concomitant anthracycline therapy. Decreasing the dose or discontinuation of Herceptin and initiation with B-blockers and angiotensin-converting enzymes can reverse signs and symptoms of cardiotoxicity (Onitilo et al. 2014). Four approved PDL 1 check-point inhibitors (pembrolizumab, atezolizumab, avelumab, and durvalumab) treat early and advanced triple-negative breast cancer and are now routinely encountered in the perioperative period. The most common perioperative concern is adrenal insufficiency, appreciated in 10% of patients. An endocrine consultation throughout perioperative is integral to the multidisciplinary approach to monitor and adjust electrolyte imbalances and hormone replacement therapy as needed (Planes-Laine et al. 2019).

### Cell‐based immunotherapy or adaptive cell therapy (ACT)

The body has innate and adaptive cell-based immune mechanisms that complement each other. The innate is an immediate nonspecific response that recognizes foreign molecules and activates cellular defense mechanisms (neutrophils, macrophages, NK cells) to prevent the spread of threats quickly. Once activated, the innate system stimulates the long‐term cell-based or adaptive cell response (T lymphocytes and B lymphocyte). Cell‐based immunotherapy, also known as adoptive cell therapy (ACT) or chimeric antigen receptor T cell (CAR T cell), physically supplements the immune system with genetically manipulated T-cell lymphocytes to target a specific antigen. Initially developed for children with B-cell acute lymphoblastic leukemia targeting the B-cell CD19 and BCMA antigen, it now encompasses adult hematologic malignancies. They are customized to each patient. The side effect profile includes neurotoxicity, cardiomyopathy, and pancytopenia. The most life‐threatening complication is cytokine release syndrome (fever, vasodilatory shock, respiratory failure), which requires intensive care management. There are currently six FDA-approved CAR T-cell therapies (Table 5). The future of adaptive cell‐based therapy alone or in combination with other treatment modalities presents a personalized approach to optimize the antitumor effect while sparing healthy cells (Kruger et al. 2019).

**Table 5:** FDA-approved CART T-cell therapies

Name	Trade name	Antigen	Disease
Tisagenlecleucel	Kymriah	CD19	ALL, NHL
Axicabtagene ciloleucel	Yescarta	CD19	NHL, follicular lymphoma
Brexucabtagene autoleucel	Tecartus	CD19	Mantle cell lymphoma (MCL), ALL
Lisocabtagene maraleucel	Breyanzi	CD19	NHL
Idecabtagene vicleucel	Abecma	BCMA	Multiple myeloma (MM)
Ciltacabtagene autoleucel	Carvykti	BCMA	Multiple myeloma (MM)

*ALL* Acute lymphoblastic leukemia, *NHL* Non-Hodgkin lymphoma

#### Perioperative period

These patients are uncommonly present in the perioperative period except for emergency surgery and, more commonly, for vascular access. There are no standard perioperative strategies, and until more data is available, the best management is achieved by a multidisciplinary team familiar with the side effect profile of CAR T-cell therapy. In addition, corticosteroids, which are used in many perioperative protocols for postoperative nausea and vomiting and adjuvants in regional anesthesia, counteract CAR T-cell therapy’s effect due to its inhibition of T-cell function and should not be given within 30 days of treatment (Rohaan et al. 2019).

## Cancer vaccines

Cancer vaccines incorporate DNA, RNA, and viral tumor antigens to activate cellular and humoral responses to attack and destroy cancer cells while sparing healthy cells. Tumor vaccines are classified into immune cells, proteins, genetic DNA or RNA, and viral vaccines based on their content. Immune cell vaccines are patient‐derived irradiated tumor cells combined with an immunostimulant. They present an entire spectrum of tumor‐associated antigens to the patient’s immune system. In 1990, the bacillus Calmette‐Guerin (BCG) vaccine was the first FDA-approved immune cell‐derived vaccine to treat bladder cancer. Since then, tumor-derived vaccines have been tested in many cancers with promising results. Sipuleucel‐T (Provenge) was FDA approved in 2010 for prostate cancer patients who are no longer responsive to hormone therapy (Lollini et al. 2006).

### Perioperative period

The perioperative period is a state of immunosuppression characterized by decreased T-cell proliferation and impaired NK cytotoxicity. Two studies present a therapeutic opportunity (Bakos et al. 2018; Jarahian et al. 2009). The production of natural killer cells (NK) and CD8 T‐cell directed at tumor antigens increased, reducing tumor burden. Viral spread to staff taking care of patients is a concern and imposes delivery challenges. Side effects of Sipuleucel are mild, but more severe allergic reactions, stroke, and meningitis with epidural analgesia do occur. Currently, there are no standard practices for cancer vaccine deliveries in the perioperative period. Ongoing clinical trials will assess the safety and efficacy of implementing cancer vaccines in the perioperative period and survival outcomes.

## Oncolytic viruses

Oncolytic viruses are immune‐oncology drugs engineered to replicate and enhance the immune system to attack and destroy tumor‐selective cells. Historically, live viruses treated and eradicated various diseases, notably the Egypt 101 West Nile virus. Severe toxic reactions were noted, especially in immunocompromised patients. The advent of genetic engineering cultivated live replicating viruses, targeting entry points into the cancer cell. These newly designed viruses have several added functions. They can target the cancer cell’s transcription process, have a reporter gene that checks the pharmacokinetics of virotherapy, act as immunomodulators, and enhance cytotoxic activity (Maroun et al. 2017). To date, there are only three oncolytic viruses approved. These include Rigvir (Riga virus since 2004) for the treatment of melanoma in Latvia (Alberts et al. 2016), Oncorine (H101since 2005) for use in combination therapy for the treatment of head and neck cancers in China (Liang 2018), and talomogene (T‐VEC or Imlygic) (Liu et al. 2003), a genetically modified herpes virus, approved by the Food and Drug Administration in the USA in 2015 for the intralesional treatment of unresectable metastatic melanoma and brain tumors. These do not affect visceral metastatic lesions. Clinical trials are ongoing for combination therapy with chemotherapeutic agents, check-point inhibitors, or radiation therapy to treat metastatic disease.

The tumor microenvironment is essential in contributing to metastatic disease. Surgical stress in the perioperative period induces suppression of cellular immune response. This response primarily affects the cytotoxic activity of NK cells. The application of oncolytic viruses in the perioperative period increases the NK cell’s activity, confirming a role in preventing metastatic disease progression. The timing of treatment with oncolytic viruses is an essential factor. A single dose of the vaccinia virus before metastatic liver resection resulted in a lower disease progression rate in patients with metastatic colorectal cancer. Genetic copies were found in the resected tumor, suggesting viral targeting of cancer but not in normal liver tissue (Russell and Peng 2018).

### Perioperative period

Oncolytic viral therapy administered in the perioperative period prevents surgery‐induced immunosuppression of NK cells and improves survival. Talomogene (T-VEC) produces cytokines to boost the immune system, and flu-like symptoms or more severe tumor lysis syndrome can occur. Despite promising results, there are no standard perioperative cancer therapies to prevent disease progression. Feasibility and safety remain barriers to implementing oncolytic viral treatment in perioperative (Tai et al. 2013).

## Radiation therapies

X‐ray innovation by Wilhelm C. Roentgen in 1895 began the advent of many changes in the diagnosis of diseases and cancer treatment. Radiation therapy damages the double-stranded DNA, preventing cancer cells from replicating and leading to cell death. Cancer cells continue to die for weeks and months after radiation therapy. In addition, immunosuppression by CD8 T‐cell-mediated cytotoxicity at distant tumor sites results in visible disease regression. This systemic effect, coined in 1953, is the “abscopal effect” (Wargo et al. 2015; Brix et al. 2017).

By the early 2000s, advances in radiation delivery devices delivered high doses of energy deep into the tissues sparing the skin and surrounding areas. Innovations of sophisticated computer technologies such as 3D conformal radiotherapeutic devices (stereotactic radiation therapy), adaptive radiotherapy (ART), and image‐guided radiotherapy (IGRT) expand treatment options (Schwartz et al. 2012). Two types of radiation treatment beams exist; electromagnetic sources use X‐ and gamma rays, and charged particles consist of photons, electrons, and protons. The most common external beam radiation therapy comes from photons. They reach deep tumors in the body and scatter radiation, damaging healthy tissues. Proton beams do not scatter radiation along their travel path. They deliver a high radiation dose deep into the body while sparing normal tissue. This therapy is desirable, but the expense and size of the machines limit their use. Electron beams cannot travel very far and treat superficial tumors.

Electromagnetic sources use an external beam or internally delivered radioactive treatments in a solid form, known as brachytherapy (seed implants) or liquid (molecular radiotherapy), administered orally or via an intravenous line. These latter forms of internal radiotherapies emit radiation for some time.

### Perioperative period

Remote locations, the hazard of ionizing radiation to staff, limited patient access, and airway management can be challenging. Staff protection and hospital radiation safety measures are paramount.

The preoperative evaluation focuses on identifying comorbidities and complications from radiation treatment. The systemic effects are often seen early, including fatigue, cognitive dysfunction, and anorexia. Later complications of radiation therapy occur at the cellular level through an innate cellular immune response where the normal tissue repair process is deregulated and fibrosis forms. These chronic changes arise over time and are most apparent in head and neck cancer patients — decreased range of motion, airway rigidity, and trismus. Difficult mask ventilation and laryngoscopy present considerable airway management challenges. Cardiac disease is also seen many years after treatment and necessitates a preoperative cardiology evaluation.

External beam therapy does not usually require anesthesia unless patient discomfort due to positioning is prohibitive. The brachytherapy suite is located off‐site on the primary surgical floor. It is an intricate and complex operating room. The goal of anesthesia is sedation, analgesia, and immobility. Sedation, regional, and general anesthetic management are all acceptable. Intraoperative radiation therapy (IORT) delivers radiation to the tumor margins immediately after surgical resection while the patient is under general anesthesia. This modality reduces treatment time and minimizes radiation exposure to healthy tissues (Gourkanti et al. 2018). Prolonged anesthesia time and limited patient access during radiation treatment require detailed preoperative planning. In addition, chronic radiation changes make the tissues more friable, leading to increased intraoperative bleeding. The placement of an arterial line for continuous blood pressure monitoring and frequent blood sampling is appreciated. Difficult intravenous placement often requires ultrasound-guided peripheral vascular and central venous access.

Chronic problems associated with fibrosis can limit an individual’s tolerance to treatment (Mancini and Sonis 2014). Consequently, radiation therapy is often combined with other treatment modalities to decrease the toxicity and duration of treatment preoperatively. Traditionally, chemo- and radiation-combined therapy have been successful. Combining immunotherapy treatments with radiation therapy is an area of great interest. The premise rests in radiation therapy’s immune‐modulatory effects, enhancing immunotherapy’s therapeutic effect for improved cancer survival (Ridolfi et al. 2014).

## Novel interventional radiology procedures for cancer

The trend towards minimally invasive procedures and novel technologies in interventional radiology gives patients more options for nonsurgical treatment of cancer conditions.

Almost all patients will come across the interventional radiology suite at some point in their treatment. Complex minimally invasive procedures to treat cancer using radioactive particles (photons, protons, electrons), heat (microwave ablation), cold (cryoablation), electric current (radiofrequency ablation), and embolization guided by imaging modalities such as ultrasound, x‐ray, CT, MRI, and fluoroscopy in a nonoperating room type setting continue to grow. Creativity to combine different treatment modalities evolves. A more complicated treatment combines a high dose of chemotherapy and transarterial embolization (TACE) or ablation or radiation therapy as an alternative to standard therapy or surgical resection (Guan et al. 2012). TACE remains the gold standard for hepatocellular cancer and is an increasing modality in other cancer treatments.

### Perioperative period

Preoperatively, patient triage minimizes adverse events and improves patient outcomes. Abnormal liver function tests, severe comorbidities, and risk of bleeding from prior transjugular intrahepatic portosystemic shunt (TIPS) or untreated esophageal varices at high risk of bleeding can be prohibitive. Preoperative octreotide is given to patients with a history of carcinoid cancer to minimize acute hormone release‐carcinoid crisis. Patients taking bevacizumab, a monoclonal antibody used to treat various cancers, should discontinue its use 4–6 weeks before TACE therapy. Bevacizumab is associated with increased sepsis, thromboembolic events, and delayed wound healing. Routine antibiotics are not needed. However, in any biliary abnormality, antibiotics decrease the risk of infection.

Lengthy procedures require general anesthesia and adequate intravenous and invasive monitoring depending on the extent of the procedure and patient factors. Pain is the most common clinical symptom after the TACE procedure, often extending hospital stays and reducing patient willingness for further treatments. Lidocaine infusion given before or between chemotherapy has decreased postoperative pain.

Post‐procedure ischemic changes to the liver initiate a post‐embolization syndrome. Clinical findings such as fever, nausea, upper right quadrant pain, ileus, and increased liver function test are present in up to 80% of patients and last 3–4 days. Postoperative care includes hydration, normovolemia, antiemetics, and steroid administration (Gupta et al. 2003). Postoperatively, patients are admitted for overnight observation or discharged home if criteria are met.

Advancements in technologies and safety in performing procedures propel novel image-guided therapeutic interventions. Innovations guide the future of interventional radiology into the sphere of virtual reality. For example, the operator could fuse static and real‐time images or project a 3D image to guide a flawed visualization procedure and avoid essential structures that may otherwise be invisible. Another area is robotics, which is applied successfully in the surgical disciplines. Image‐guided interventions using robotics would allow the interventional radiologist to perform procedures outside the interventional suite in the adjoining room, significantly decreasing the radiation exposure to all staff (Midulla et al. 2019).

Whatever the future holds, searching for combined modalities improves clinical outcomes in cancer patients. The growth of nonoperating room procedures presents unique challenges and opportunities. Collaboration among medical disciplines caring for cancer patients is essential. Anesthesiologists are vital team members to ensure patient safety and successful perioperative outcomes.

## Conclusion

A better understanding of the molecular profile of tumors led to new cancer treatment modalities. While hematologic, pulmonary, gastrointestinal, hepatotoxicity, and cardiac side effects are common and well‐tolerated in the short term, better understanding and management of long‐term side effects from emerging cancer therapies continue to evolve. Some patients may require lifelong treatment, and the impact on quality of life poses an increasing challenge to the patient and the perioperative physician.

## Data Availability

Data sharing does not apply to this article as no datasets were generated or analyzed during the current study.

## Abbreviations

5FU: 5‐Fluorouracil
DLCO: Carbon monoxide diffusion capacity
NSAIDs: Nonsteroidal anti‐inflammatory drugs
AKI: Acute kidney injury
CKD: Chronic kidney disease
HIPEC: Hyperthermic intraperitoneal chemotherapy
CDC: Complement‐dependent cytotoxicity
ADCC: Antibody‐dependent cell‐mediated cytotoxicity
NK: Natural killer cells
HER2: Human epidermal growth factor 2
ACTH: Adrenocorticotropic hormone
HbA1c: Hemoglobin A1c
ACT: Adoptive cell therapy
TIL: Tumor‐infiltrating lymphocytes
TCR: T-cell receptors
CAR: Chimeric antigen receptors
BCG: *Bacillus* Calmette‐Guerin
H101: Oncorine
T‐VEC: Imlygic
ART: Adaptive radiotherapy
IGRT: Image‐guided radiotherapy
IORT: Intraoperative radiation therapy
ECM: Extracellular matrix
TACE: Transarterial embolization
TIPS: Transjugular intrahepatic portosystemic shunt
